# Self-Perception of the Knee Is Associated with Joint Motion during the Loading Response in Individuals with Knee Osteoarthritis: A Pilot Cross-Sectional Study

**DOI:** 10.3390/s21124009

**Published:** 2021-06-10

**Authors:** Haruki Toda, Tsubasa Maruyama, Koji Fujita, Yuki Yamauchi, Mitsunori Tada

**Affiliations:** 1 Digital Human Research Team, Artificial Intelligence Research Center, National Institute of Advanced Industrial Science and Technology, Tokyo 135-0064, Japan; tbs-maruyama@aist.go.jp (T.M.); m.tada@aist.go.jp (M.T.); 2 Department of Functional Joint Anatomy, Graduate School of Medical and Dental Sciences, Tokyo Medical and Dental University, Tokyo 113-8510, Japan; fujiorth@tmd.ac.jp; 3 Department of Orthopaedic Surgery, Dojin Hospital, Okinawa 901-2133, Japan; yamauchi.orth@tmd.ac.jp

**Keywords:** Fremantle Knee Awareness Questionnaire, gait, inertial measurement unit, joint motion, motion capture, osteoarthritis, self-perception

## Abstract

Small knee flexion motion is a characteristic of gait in individuals with knee osteoarthritis. This study examined the relationship between knee flexion excursion in loading response and knee self-perception in individuals with knee osteoarthritis. Twenty-one individuals with knee osteoarthritis participated in this study. Knee flexion excursions in loading response while walking at a comfortable and a fast-walking speed were measured using an inertial measurement unit-based motion capture system. The degree of knee perceptual impairment was evaluated using the Fremantle Knee Awareness Questionnaire (FreKAQ). The relationships between the FreKAQ score and gait variables and knee function were evaluated by calculating the correlation coefficient. The unique contributions of knee self-perception and muscle strength to knee flexion excursion in loading response were analyzed using hierarchical linear regression. Knee self-perception was significantly correlated with pain during walking, muscle strength and knee flexion excursion at fast speed. In the fast speed condition only, impaired knee self-perception was inversely proportional to knee flexion excursion and accounted for 21.8% of the variance in knee flexion excursion. This result suggests that impaired self-perception of the knee may help to explain the decrease in the knee flexion excursion in the loading response in individuals with knee osteoarthritis.

## 1. Introduction

Knee osteoarthritis (OA) is one of the most common musculoskeletal disorders among older adults [1]. Many individuals with knee OA experience clinical symptoms such as pain, articular stiffness and deformation, restriction of range of motion (RoM), and muscle weakness [2]. These contribute to abnormal knee joint motions that are often observed in individuals with knee OA during walking [3,4]. 

In addition, pain and anatomical changes to knee joint soft tissues by osteoarthritis also induced impaired tactile acuity and proprioceptive accuracy due to articular mechanoreceptor impairment, weakness of muscle-spindle sensitivity, and knee joint inflammation [5,6,7]. A neuroimaging study revealed that knee representation was reorganized in the brain cortex of individuals with knee OA [8]. This reorganization in the brain cortex impacts self-perception [9], which is utilized to produce motor programs [10]. Distortion of the self-perception of the knee has been reported to affect knee-related disability [11] and may affect the abnormal knee joint motion during walking. However, to our knowledge, no study has examined the relationship between self-perception of the knee and knee joint motion.

Knee flexion motion can attenuate the impact load on the articular cartilage during weight acceptance. Thus, a previous study of knee flexion motion in the loading response was focused on the biomechanical change of the knee during gait in individuals with knee OA [12]. Individuals with knee OA demonstrated smaller knee flexion excursion during the loading response of gait than healthy individuals [13]. Small knee flexion excursion is typically accompanied by increased muscle co-contractions around the knee to increase dynamic joint stability [14]. Muscle co-contractions contribute to increasing joint contact force [15]. A higher joint contact force may put the joint at risk for progressive cartilage degeneration [16]. Individuals with knee OA strategically reduce the external forces generated in the knee joint by reducing the joint angle [12]. In the long run, if the small knee flexion excursion is controlled by co-contraction, increasing the compressive load may accelerate the progression of cartilage degeneration [17]. Additionally, in daily life, people need to walk at various speeds depending on their situation and environment. When walking faster, individuals with knee OA are more likely to experience increased mechanical stress on their knee joint [18]. Consequently, knee flexion excursion in the loading response was important for shock attenuation of the knee joint in patients with knee OA, while walking at both comfortable and fast walking speeds.

Knee flexion motion in the loading response is accomplished by generating tension force through quadriceps contraction. Although individuals with knee OA have muscle weakness in the quadriceps [19], a relatively low-level eccentric quadriceps contraction is required for generating knee flexion during walking [20]. In contrast, alterations in sensory perception in both the central and peripheral nervous systems by knee OA may affect knee joint motion during walking as well as quadriceps muscle weakness. Sensory perception contributes to appropriate limb motion and joint positioning as a feedforward and feedback system. Impaired sensory perception contributes to the ineffectively preparation for the impact and loading during weight acceptance [21]. Thus, joint stability was increased by co-contraction around the joint and keeping a stable joint position. The knee joint is more stable in the extended position during walking [20]. In contrast, this strategy discourages movement that is adapted to the external and internal environment [22]. Bennell et al. [3] found that a decreased joint position sense of the knee correlates with less knee flexion at the initial contact for joint stability. Therefore, we need to focus on both motor and sensory-perceptual aspects when considering small knee flexion excursion in the loading response during walking in individuals with knee OA.

We, therefore, aimed to investigate the relationship between knee joint excursion in the loading response while walking at a comfortable and fast walking speed and the self-perception of the knee in individuals with knee OA. We hypothesized that impaired self-perception of the knee relates to a reduction of knee flexion excursion in the loading response in both comfortable and fast speed conditions.

## 2. Materials and Methods

### 2.1. Participants

Participants with knee OA were recruited consecutively at two hospitals. Twenty-one people with knee OA were included in this study. The eligibility criteria included the following: (1) a diagnosis of tibiofemoral knee OA, evaluated using weight-bearing anteroposterior radiographs in addition to the clinical symptoms, e.g., knee pain, stiffness and swelling, and (2) the ability to walk independently without any ambulatory assistive devices. If participants had bilateral knee OA, the limb comprising the more symptomatic knee was selected for this study. The exclusion criteria were as follows: (1) history of knee surgery, (2) rheumatoid arthritis, (3) neurological disorder, and (4) reduced ability to walk (walking speed <1.0 m/s at comfortable walking speed). This study was conducted according to the Declaration of Helsinki, and the experimental protocol was approved by the local institutional review board (M2018-123). All participants gave written informed consent before the measurements were taken.

### 2.2. Clinical Evaluation

The Kellgren–Lawrence (K-L) grade for knee OA severity and femorotibial angle (FTA) were assessed from weight-bearing anteroposterior radiographs [23]. Knee joint pain during walking was assessed using the visual analog scale (VAS). We evaluated the degree of knee perceptual impairment using the Fremantle Knee Awareness Questionnaire (FreKAQ) [24], which comprises nine items investigating neglect-like symptoms, reduced proprioceptive acuity, and perceived body shape and size scored from 0 (more knee awareness) to 36 (less knee awareness) (Table 1). The FreKAQ was developed by modifying Fremantle Back Awareness Questionnaire (FreBAQ) that has a theoretical construct of perceptual impairment of back [25]. The person reliability, Cronbach’s alpha, and intraclass correlation coefficient of the FreKAQ were 0.81, 0.88, and 0.76, respectively [24]. In addition, the validity of the questionnaire was supported by the association with knee pain intensity, disability, pain catastrophization, kinesiophobia, and anxiety [24]. The VAS and FreKAQ scores were evaluated using a self-reporting questionnaire.

### 2.3. Range of Motion (RoM)

The passive RoM of knee extension was measured using a standard goniometer (OG wellness Co., Ltd., Okayama, Japan) in 5° increments. This measurement was taken on an examination table with the participant in a supine position, exhibiting maximal knee extension. The goniometer was aligned over the sagittal axis of the thigh and lower leg.

### 2.4. Muscle Strength

The maximum isometric knee extension strength was measured using a hand-held dynamometer (μTas F-1; Anima Corp., Tokyo, Japan) following previously validated protocols [26]. This measurement was taken on an examination table with the participant in a sitting position, the hip and knee joints at a 90° flexion, lower leg perpendicular to the floor, and feet not touching the floor. The sensor of the hand-held dynamometer was placed in front of the lower leg, directly proximal to the ankle during extension. All participants performed two maximal extensions for 5 s after a few practice trials. The maximum value between the two extensions was used for the analyses. Each strength value and lever arm were converted into a torque ratio for body weight (Nm/kg).

### 2.5. Gait Measurement

**Figure 1:**
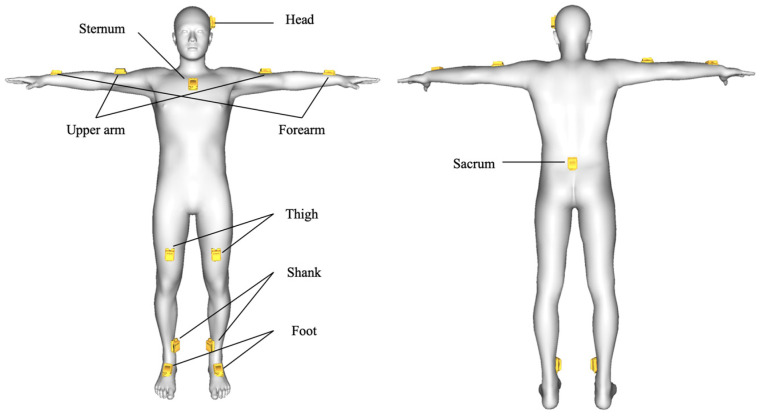
Attachment position of inertial measurement units.

An IMU-based MoCap was used to measure the joint angle of the whole body. Thirteen IMUs (MTw; Xsens Technologies Inc., Enschede, Netherlands) were attached to the participant’s head, sternum, sacrum, and to the two upper arms, forearms, thighs, shanks, and feet (Figure 1). Participants were allowed to use their own shoes to walk to ensure high fidelity in the measurement of the natural function of the knee [13]. Before measurements, IMU orientations relative to the corresponding body segments were determined using a reference pose (i.e., T-pose) for calibrating the motion capture system.

The participants walked approximately 12 m on a walkway in the hospital under the two walking speed conditions: comfortable and fast. For the fast-walking speed condition, participants were instructed to walk as fast as possible, but not run. The order of the walking sessions was randomized.

We calculated the joint angles using a posture-reconstruction plugin [27] running on DhaibaWorks—a self-developed motion analysis software [28]. The full-body motion was reconstructed by combining the IMU orientation data and the individual body model with a link structure. To estimate the body model dimensions based on the database of Japanese body dimensions [28], the participant’s body height and weight measurements were used. In addition, the FTA and passive knee extension RoM of the participant was reflected in the body model. The motion data were sampled at 60 Hz. The validation of the knee flexion angle and stride length relative to those measured using optical MoCap were previously evaluated using coefficient of determination and defined as very strong (R^2^ ≥ 0.64), with confirmed values to of 0.82 (knee), and 0.86 (stride length) [29].

**Figure 2:**
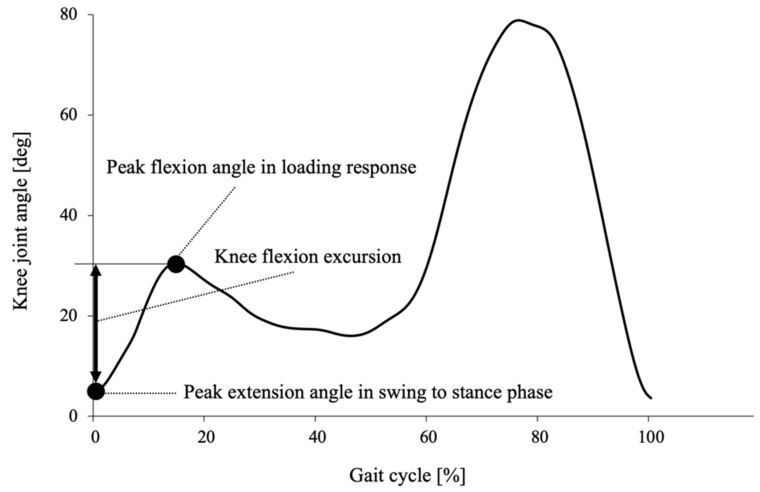
Example of time series data of the knee flexion angle in one gait cycle of a subject. Black dots indicate the values extracted for calculation of knee flexion excursion in loading response. Knee flexion excursion in the loading response was calculated from the amplitude of displacement between the peak extension angle at the initial contact and peak flexion angle in the loading response.

The motion data were digitally filtered using a Butterworth low-pass filter with a cut-off frequency of 6 Hz. We defined the beginning of a gait cycle (initial contact) as the point of peak knee extension angle in a swing to stance phase. The knee flexion excursion in the loading response was calculated from the displacements between the peak extension angle and peak flexion angle in the loading response (Figure 2). Data for five strides during steady-state walking were extracted. The stride length was calculated using the distance of the feature point at the heel. Walking speed was calculated using the stride length and time. The average of the values calculated from the five gait cycles was used for analysis.

### 2.6. Statistical Analysis

The Shapiro–Wilk test was used to check data distribution. A paired t-test and Wilcoxon signed-rank test were performed to analyze the difference in the walking speed, stride length, and knee flexion excursion in loading response between comfortable and fast speed conditions. The minimal detectable change (MDC) was calculated using the relation: SEM × 1.96 × √2 [29]. The MDC indicates the minimum amount of change that is not likely to be due to an accidental error in measurement [30]. Pearson and Spearman correlation coefficients were used to examine the relationships between variables.

We performed a three-block hierarchical multiple linear regression to identify the knee function associated with knee flexion excursion in each speed condition. We chose this analysis to determine the unique contribution of muscle strength and self-perception of the knee to the total variance explained by the model. At first, age, K-L grade, VAS walking score, and walking speed in each condition were entered in a basic model as confounding variables into the independent variables using forced entry because previous studies reported that these variables affected the knee joint motion during walking [15,31,32,33]. After that, the muscle strength, which is considered to be a major contributor to the control of the knee flexion in the loading response, and FreKAQ score were entered in addition to the basic model using forced entry, respectively. 

Statistical significance was accepted for *p* values of <0.05. All data were analyzed using the SPSS version 25.0 statistical software (SPSS Japan Inc., Chicago, IL, USA).

## 3. Results

Table 2 shows the characteristics of the participants. As shown in Table 3, walking speed across the two conditions was successfully modulated, with increased speed reflected in the conditioning effects. Compared to the comfortable speed condition, the fast condition was 21% faster. As the walking speed increased, stride length and knee flexion excursions significantly increased. The FreKAQ score correlated significantly with VAS walking score, muscle strength and knee flexion excursion at fast speed (Table 4).

**Figure 3:**
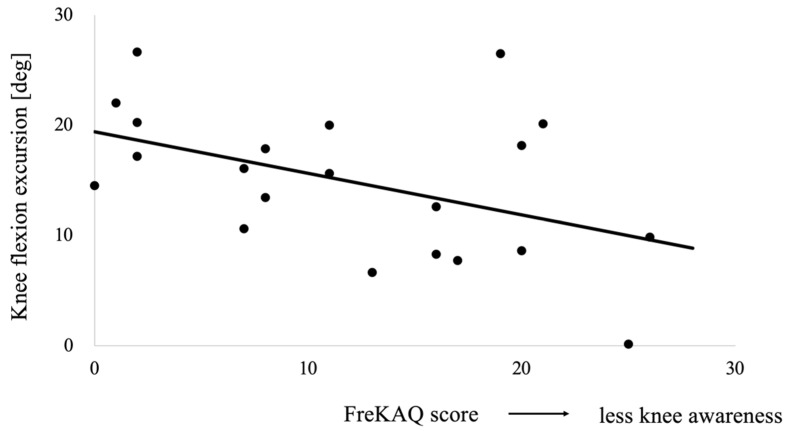
The relationship between the knee flexion excursion in loading response at fast speed and the Fremantle Knee Awareness Questionnaire (FreKAQ) score. High FreKAQ score indicates less knee awareness.

Hierarchical multiple regression analysis results are indicated in Table 5. In the comfortable speed condition, when adding muscle strength and FreKAQ score to the models, the R2 values did not improve significantly. In the fast speed condition, by entering FreKAQ score, the R2 value significantly improved, and the model became statistically significant. FreKAQ score correlated negatively with knee flexion excursion (Figure 3) and accounted for 21.8% of the variance in this excursion after controlling for the effect of the covariates during fast walking. However, muscle strength was not determined to be a significant factor.

## 4. Discussion

This study examined the relationship between knee self-perception and knee flexion excursion in the loading response during walking in individuals with knee OA. We found that an impaired self-perception of the knee related to a reduction of knee joint excursion in the loading response while walking at a fast speed. This result partially supported our hypothesis.

Our results revealed that knee OA patients with an impaired self-perception of the knee demonstrated smaller knee flexion excursion at fast walking speed. The FreKAQ score explained 21.8% of variance in the knee flexion excursion at fast walking speed after controlling for the effect of age, K-L grade, VAS, and walking speed. One previous study reported a relationship between joint kinematics during walking and an impaired sensory caused by knee OA. Bennell et al. [3] reported the relationship between knee joint angle at the initial contact and knee joint position sense in individuals with knee OA. The joint position sense is transmitted by afferent nerves from the articular mechanoreceptor and muscle-spindle to the sensory cortex [34]. Thus, the function of peripheral mechanoreceptors is important. In contrast, our study focused on the self-perception of the knee evaluated by the FreKAQ developed by modifying FreBAQ [25]. This questionnaire was validated by pain catastrophizing, kinesiophobia and anxiety [24]. Our study showed that FreKAQ score was significantly associated with pain during walking. Similarly, Nishigami et al. [24] found that the FreKAQ score correlated significantly with pain intensity during motion in individuals with knee OA. Pain contributes to body perception disturbance [35]. The FreKAQ score reflects a pain-related distortion in the self-perception of the knee in the central nervous system.

The FreKAQ investigated neglect-like symptoms, proprioceptive acuity, and perceived body shape and size [24]. Individuals with knee OA were previously shown to have a high FreKAQ score and large perceived knee size [36]. The integration of sensory-perceptual information in the motor and sensory cortex is related to these elements [34]. The information about body perception was utilized to produce motor programs in the supplementary motor area (SMA) [10]. Anticipatory movements are contingent on the feedback information of perception as the body’s awareness of position and movement in space [21]. When walking at a comfortable speed, lower leg movements are automatically produced by central pattern generators in the spinal cord [10]. In contrast, when walking faster, activities in the SMA increase to control lower leg movements corresponding to increased walking speed [37]. However, the disturbance of self-perception reduces SMA activation [38]. We speculated that because individuals with impaired self-perception due to knee OA had difficulty in producing joint movements corresponding to increasing walking speed, their knee flexion excursion seemed to be smaller in the loading response at fast walking speeds.

Knee flexion excursion in the loading response was accomplished by eccentric quadriceps contraction [39]. The relationship between the degree of knee flexion excursion and maximum muscle strength of the quadriceps has been reported previously [3,40,41,42]. However, this study found that knee flexion excursion was not related to the muscle strength of the quadriceps in participants with knee OA. Lewek et al. [39] and Farrokhi et al. [40] examined patients with patellofemoral osteoarthritis and anterior cruciate ligament injury and reconstruction, respectively. However, these diseases are different from tibiofemoral knee OA in terms of the impairment of the knee extension mechanism. While the participants in the study by Murray et al. [41] had knee OA, they also had large body weights (over 100 kg) and high body mass indices (mean 34.62 kg/m^2^). Obesity is associated with neuromuscular adaptations, leading to reduced knee joint loads during gait [42]. Therefore, the results of these studies may not be applicable to our participants with tibiofemoral knee OA and standard body size. Although Bennell et al. [3] also reported a relationship between knee flexion excursion and the maximum muscle strength of the quadriceps in patients with knee OA, their correlation coefficient was very small (r = 0.17). Relatively low-level eccentric quadriceps contraction is required for generating knee flexion during walking [20]. It may be challenging to determine whether the degree of knee flexion excursion was related to the maximum isometric muscle strength of the quadriceps.

Individuals with knee OA demonstrated small knee flexion excursion to stabilize the knee joint and reduce joint torque [12,14]. In constant, when walking faster, they are more likely to experience increased mechanical stress on their knee joint [18]. Thus, shock attenuation by knee flexion motion is more important at a fast-walking speed than a comfortable speed. Generally, the knee flexion angle in loading response was positively related to walking speed [33]. In this study, walking speed, stride length, and knee flexion excursion were all significantly increased at fast walking speed. However, the difference in the knee flexion excursion between comfortable and fast speeds was smaller than its MDC although the differences in walking speed and stride length were larger than their MDC. Nevertheless, in this study, the self-perception of the knee related to the knee flexion excursion while walking at fast-walking speed.

There are some limitations to this study. First, the study employed a cross-sectional design. Therefore, we cannot identify a causal relationship between knee self-perception and knee flexion excursion from the results of this study. Second, a relatively high number of female participants were involved in this study, possibly causing a selection bias. As sex differences in walking patterns [43] and muscle strength [44] have been reported, the generalization of our results is questionable. Third, the IMU-based MoCap system employed during this study has not been used previously to evaluate the kinematic profiles of healthy elderly individuals while walking. Thus, it is unclear whether the knee flexion excursion of the participants in this study is less than that of healthy individuals. Finally, this study should be considered as an exploratory study with a small sample size although the number of participants in the present study exceeded the minimum number of cases per variable (of at least two) reported in a previous study [45].

## 5. Conclusions

This study examined the relationship between self-perception of the knee and knee joint excursion during the loading response when walking at comfortable and faster speeds in 21 individuals with knee OA. Our pilot cross-sectional study suggested that the impaired self-perception of the knee correlated with a reduction of knee joint excursion in the loading response while walking at a fast speed. Indeed, self-perception of the knee accounted for 21.8% of the variance in the knee flexion excursion while walking at a fast speed. The impaired self-perception of the knee may help to explain the decrease in the knee flexion excursion in the loading response while walking at a fast speed in individuals with knee OA.

## Figures and Tables

**Table 1:** Items of the FreKAQ ^1^.

Item
I feel like my knee is not part of my own body
2. To move my knee the way I want to, I feel like I have to concentrate all my nerves there
3. Sometimes I feel like my knee moves without any connection to what I intend it to do
4. When performing activities of daily living (housework, work, etc.), I do not know how much my knee is moving
5. When performing activities of daily living (housework, work, etc.), I do not know what kind of position my knee is in
6. I cannot image my knee’s contour correctly
7. I feel like my knee is bigger (swollen)
8. I feel like my knee has shrunk
9. My knee feels differences with right and left. (One side feels dull or fat)

^1^ Fremantle Knee Awareness Questionnaire.

**Table 2:** Characteristics and outcome measures of participants.

Characteristics, *n* = 21	
Age (years), mean (SD)	72.1 (7.9)
Sex, *n* (%)	
Male	4 (19.0%)
Female	17 (81.0%)
Height (m), mean (SD)	1.54 (0.94)
Body weight (kg), mean (SD)	59.5 (9.2)
BMI ^1^ (kg/m2), mean (SD)	24. 9 (3.4)
K-L grade ^2^, *n* (%)	
Grade I	2 (9.5%)
Grade II	7 (33.3%)
Grade III	7 (33.3%)
Grade IV	5 (23.8%)
VAS ^3^ walking (mm), mean (SD)	35.4 (33.7)
Muscle strength (Nm/kg), mean (SD)	1.06 (0.28)
FreKAQ ^4^, mean (SD)	12. 0 (8.1)

^1^ Body mass Index. ^2^ Kellgrens-Lawrence grade. ^3^ Visual analog scale. ^4^ Fremantle Knee Awareness Questionnaire. SD: Standard deviation.

**Table 3:** Comparison results in walking speed, stride length and knee flexion excursion between speed conditions.

	Comfortable	Fast	MDC ^1^	*p*-Value
Walking speed (m/s), mean (SEM)	1.23 (0.05)	1.51 (0.07)	0.15	0.0001
Stride length (m), mean (SEM)	1.15 (0.02)	1.25 (0.02)	0.05	<0.0001
Knee flexion excursion (deg), mean (SEM)	12.1 (1.34)	14.8 (1.46)	3.73	0.0002

^1^ Minimal detectable change. SEM: Standard error of measurement.

**Table 4:** Correlation matrix between all variables.

	1	2	3	4	5	6	7	8
1. Age								
2. K−L grade ^1^	0.171							
3. VAS ^2^ walking	0.142	0.349						
4. Muscle stregth	−0.164	−0.370	−0.125					
5. FreKAQ ^3^	0.237	0.350	0.467 *	−0.469 *				
6. Walking speed (Comfortable)	0.290	−0.205	−0.181	0.186	−0.182			
7. Knee flexion excursion (Comfortable)	0.244	−0.463 *	−0.230	0.236	−0.361	0.191		
8. Walking speed (Fast)	−0.163	−0.306	−0.301	0.445 *	−0.432	0.710 *	0.051	
9. Knee flexion excursion (Fast)	0.063	−0.461 *	−0.209	0.277	−0.458 *	−0.017	0.915 *	0.005

*: *p* < 0.05. ^1^ Kellgren-Lawrence grade. ^2^ Visual analog scale. ^3^ Fremantle Knee Awareness Questionnaire.

**Table 5:** Results of hierarchical regression analysis predicting knee flexion excursion in loading response.

Condition	Independent Variable	R^2^	ΔR^2^	ΔF	*p*-Value	B	95% CI ^1^	β
Comfortable speed	Covariates	0.457	0.457	3.371	0.035			
	+ Muscle strength	0.475	0.018	0.502	0.489	3.209	−6.443, 12.86	0.148
	+ FreKAQ ^2^	0.546	0.089	2.943	0.107	−0.319	−0.715, 0.077	−0.421
Fast speed	Covariates	0.341	0.341	2.073	0.132			
	+ Muscle strength	0.392	0.051	1.256	0.280	6.192	−23.46, 10.757	0.261
	+ FreKAQ ^2^	0.559	0.218	7.384 *	0.016	−0.543 *	−0.969, −0.117	−0.659

*: *p* < 0.05. Covariates include age, K-L grade ^3^, VAS ^4^ walking, and walking speed. R^2^ is unadjusted explained variance. ΔR^2^ is change in explained variance from the first step. B is the standardized regression coefficient. Β is the unstandardized regression coefficient. ^1^ Confidence interval. ^2^ Fremantle knee awareness questionnaire. ^3^ Kellgren-Lawrence grade. ^4^ Visual analog scale.

## Data Availability

Data sharing not applicable.
